# CT-Based Hounsfield Units for Pre-donation Liver Steatosis Assessment: Enhancing Transplant Outcomes and Efficiency

**DOI:** 10.7759/cureus.61196

**Published:** 2024-05-27

**Authors:** Jeffrey Campsen, Carrie Poole

**Affiliations:** 1 Surgery, University of Utah School of Medicine, Salt Lake City, USA; 2 Organ Procurement Organization, Donor Connect, Salt Lake City, USA

**Keywords:** liver transplant surgery, liver steatosis, deceased organ donation, orthotropic liver transplant, diagnostic value of ct scan, fatty liver disease, hounsfield unit (hu)

## Abstract

Steatotic liver grafts are associated with increased post-transplant complications and graft failure. The field of transplantation faces a challenge in the absence of a reliable pre-donation protocol for quantitatively assessing steatosis in cadaveric liver grafts. Current pre-donation evaluation protocols often involve non-contrast computed tomography (CT) scans of the chest and/or abdomen as an initial step in organ donation assessment. These routine scans have the potential to identify and quantify hepatic fat content when more than 20% of the liver parenchyma is affected. By incorporating both abdominal and thoracic CT scans during the donor workup, an assessment of the quality of the liver and spleen can be achieved. Our study is based on the hypothesis that a precise pre-donation evaluation utilizing Hounsfield units (HU) derived from CT images of the liver and spleen can provide transplant programs with crucial data regarding the extent of steatosis. This approach is envisioned as a significant advancement that could potentially eliminate the need for preoperative liver biopsies by offering essential information to streamline the evaluation process.

## Introduction

The escalating prevalence of end-stage liver disease, coupled with the critical shortage of viable deceased donor livers, presents a significant challenge to modern transplant medicine. This discrepancy has compelled transplant centers to reevaluate and broaden the criteria for acceptable grafts, including those with varying degrees of steatosis [1]. Steatotic grafts, historically associated with increased post-transplant complications and graft failure, result from hepatic fat content exceeding 5-10%. Despite the growing demand, the field of transplantation faces a lack of a reliable pre-donation protocol for quantitatively assessing steatosis in cadaveric liver grafts. Current practice often involves utilizing non-contrast computed tomography (CT) scans of the chest and/or abdomen as an initial step in organ donation evaluation. These scans have the potential to detect and quantify hepatic fat involvement when at least 20-33% of the liver parenchyma is affected, demonstrating a sensitivity and specificity of 85 and 100, respectively [2]. The incorporation of both abdominal and thoracic CT scans during the donor workup inadvertently offers insights into the condition of the liver and spleen. Based on the premise that a precise pre-donation evaluation utilizing Hounsfield units (HU) derived from CT images of the liver and spleen can provide transplant programs with essential data on the extent of steatosis, our study posits this approach as a significant advancement. This critical information has the potential to streamline the need for preoperative liver biopsies. Moreover, accurately determining the degree of steatosis empowers transplant centers in their decision-making process regarding resource allocation for graft preparation tailored to the recipient, thereby reducing costs, minimizing organ discards, and ultimately, saving lives.

## Materials and methods

Study data were obtained from the United Network for Organ Sharing (UNOS) database of individuals evaluated for deceased donation between January 1, 2021, and January 1, 2023, by the Organ Procurement Organization (OPO), Donor Connect. Inclusion criteria encompassed patients who underwent a liver biopsy with pathology evaluation and had a non-contrast CT scan including liver and spleen images accessible for review by a transplant surgeon. Data collection utilized UNOS reports, chart reviews, UNOS donor data, and electronic medical record reviews. The study received ethical approval from the University of Utah Institutional Review Board (approval IRB_00148714).

Evaluation of donors

Non-contrast abdominal and/or chest CT scans with accessible images were reviewed by transplant surgeons and radiologists to assess liver and spleen HU (HUL and HUS). Liver biopsies were conducted before or after donation but before transplant. Pathology reports defined the degree of hepatic steatosis (%macro/micro/total); the original slides were unavailable for additional review. HUL/HUS ratios and the HUL-HUS delta between organs were calculated.

Definitions

Biopsy diagnostic criteria for steatotic liver are defined. Steatosis severity is mild (grade 1), moderate (grade 2), or severe (grade 3) based on the percentage of fatty infiltration: <30%, 31-60%, or >61%, respectively. Macro or large droplet steatosis are fat globules larger than hepatocyte nuclei. Micro or small droplet steatosis fat globules are smaller than hepatocyte nuclei. CT diagnostic criteria for steatosis arenon-contrast thoracic/abdominal CT scans calculated HU using imaging software where 1) HUL < HUS and/or HUL < 40HU; 2) HUL/HUS < 0.8 3) HUL-HUS delta < -10HU.

## Results

During the two-year review period, a total of 375 cadaveric donors were identified within the Utah Organ Procurement Organization region. Among them, 28 donors underwent both liver biopsy and non-contrast CT scanning of the chest or abdomen prior to organ donation. Subsequently, a subset of 17 donors was selected for comprehensive analysis, encompassing liver biopsy reports and suitable non-contrast CT images for evaluation by transplant surgeons (Table 1). Notably, the decision to exclude a liver from transplantation did not exhibit a significant association with the presence of >30% steatosis as observed on biopsy results.

**Table 1:** Raw data of 17 donors HU: Hounsfield units, HUL: liver HU; HUS: spleen HU; Abd: abdomen

% Macro	% Micro	total %	CT Type	HU L	HU S	HU L/S	HU L - S	Outcome
0	5	5	AbdCT	44	37	1.1	+7	Transplanted
0	5	5	AbdCT	66	53	1.2	+13	Transplanted
5	10	15	AbdCT	58	55	1.0	+3	Transplanted
15	5	20	AbdCT	74	69	1.0	+5	Non-Utilized
10	20	30	AbdCT	59	49	1.2	+10	Transplanted
40	5	45	AbdCT	24	47	0.5	-23	Transplanted
15	0	15	AbdCT	59	34	1.7	+25	Transplanted
5	20	25	AbdCT	62	57	1.0	+5	Transplanted
30	5	35	AbdCT	39	49	0.8	-10	Transplanted
10	5	15	AbdCT	68	64	1.0	+4	Non-Utilized
50	0	50	AbdCT	32	no spleen			Transplanted
0	5	5	AbdCT	50	49	1.0	+1	Transplanted
0	5	5	AbdCT	58	53	1.0	+5	Transplanted
0	5	5	AbdCT	77	76	1.0	+1	Transplanted
0	5	5	AbdCT	65	60	1.0	+5	Transplanted
0	0	0	AbdCT	61	56	1.0	+5	Transplanted
5	0	0	AbdCT	70	69	1.0	+1	Transplanted

The donors were stratified based on the extent of macrosteatosis identified in liver biopsies, aligning this with various CT-derived parameters including HUL, HUS, and the discrepancy in HU values between the liver and spleen (delta HUL-HUS). Two distinct categories emerged from the analysis (see Table 2): 1. Less than 30% macrosteatosis: characterized by HUL >40, HUL/HUS >1.0, and delta LS >+1; 2. Greater than 30% macrosteatosis: defined by HUL <40, HUL/HUS <0.8, and delta HUL-HUS <-10.

**Table 2:** Grouping of patients by % macrosteatosis - The specific case of no spleen is highlighted as Missing/N/A for clarity and consistency - Mixed outcome is noted when the utilization of livers varied within similar parameters HU: Hounsfield units, HUL: liver HU; HUS: spleen HU

% Macro	HUL	HUS	L/S Ratio	L - S	Outcome
0	44	37	1.1	+7	Transplanted
5-15	58-74	34-69	1.0-1.7	+3 to +25	Mixed
30-50	24-39	47-49	0.5-0.8	-23 to -10	Transplanted
Missing	32	N/A N/A	N/A	N/A	Transplanted

## Discussion

Steatosis in liver grafts significantly increases the risks of primary non-function (PNF) [1], delayed graft function (DGF), biliary complications, renal impairment, prolonged hospital stays, and impacts patient and graft survival [2]. The prevalence of hepatic graft steatosis is closely linked to the rising incidence of non-alcoholic fatty liver disease (NAFLD) in the general population [3]. As NAFLD becomes more common among donors, liver graft steatosis presents an ongoing challenge in transplantation decision-making. Nevertheless, transplant programs are increasingly optimistic about their ability to mitigate the risks associated with steatotic grafts through the adoption of emerging organ preservation techniques, especially considering the growing number of deaths on transplant waitlists. An essential determinant of successful transplantation lies in the preoperative preparation that enables efficient cold ischemia time management. This factor may account for our observed discard rate, where severely steatotic livers were transplanted while mildly to moderately steatotic livers were rejected. Severe steatotic livers are commonly accepted for transplantation when transplant centers have access to timely and reliable donor information. However, the intraoperative discovery of steatosis may lead transplant centers to decline mildly to moderately steatotic livers due to the limited time available to mobilize the necessary resources for a successful transplant. Hepatic steatosis is characterized by the pattern and extent of fatty infiltration within the liver tissue. The histologic patterns of macrovesicular and microvesicular steatosis often coexist at varying degrees in the liver. Recent terminology refers to these patterns as large and small droplet fat, based on the size relationship between the nucleus and fat globule volume. Furthermore, the presence of inflammation, fibrosis, and ballooning degeneration alongside steatosis indicates significant liver injury, such as nonalcoholic steatohepatitis (NASH). Quantitatively, steatosis is graded as mild (grade 1), moderate (grade 2), or severe (grade 3) if the amount of fatty infiltration is <30%, 30-60%, or >60%, respectively [4]. The quantification of steatosis in cadaveric liver grafts lacks a standardized donor management process. While gross examination by the procuring surgeon can detect severe steatosis with some accuracy, identifying moderate or mild steatosis remains challenging. Microscopic evaluation of liver biopsies by pathologists remains the gold standard for assessing steatosis [5]. However, the detection and grading of steatosis can yield variable results due to factors like the timing of biopsy, tissue procurement method (core needle or wedge), biopsy site sampling, tissue processing techniques (e.g., frozen section vs. paraffin-embedded), and staining procedures [6]. In the US, there is a lack of uniform practices among OPOs and transplant teams regarding tissue sampling and testing, compounded by difficulties in accessing highly skilled hepatologic histopathologists. Furthermore, pre-donation liver biopsies carry inherent risks, and smaller hospitals may lack the necessary resources for comprehensive evaluations. CT of the chest and/or abdomen is commonly used in organ donation for steatosis assessment. CT imaging can detect hepatic fat involvement when around 20-33% of liver parenchyma is affected and can quantify the extent of hepatic fat accumulation with a moderate level of accuracy (sensitivity/specificity; 85/100) [7]. CT scans can also differentiate NASH from other forms of NAFLD [8,9]. Typical CT reports include measures such as liver parenchyma attenuation, liver-spleen attenuation difference, and liver-spleen attenuation ratio [10]. In a recent study involving 339 South Korean patients [11], researchers retrospectively analyzed non-enhanced CT findings and laboratory parameters. They found a significant association between the presence of hepatic steatosis and serum fasting glucose levels, triglycerides, alanine aminotransferase to aspartate aminotransferase (ALT/AST) ratio, body mass index (BMI), and hepatic steatosis index (HIS) values using various CT criteria. For instance, CT criteria for hepatic steatosis encompassed (a) an absolute liver attenuation value <48 HU, (b) a liver-to-spleen attenuation ratio <0.8, and (c) an attenuation difference between the liver and spleen <-10. NAFLD, the primary cause of steatosis, is recognized as an independent cardiovascular risk factor. Both epicardial fat thickness and liver steatosis, as ectopic lipid accumulations, are features of the metabolic syndrome [12]. Attempts have been made to establish an association between epicardial fat thickness and NAFLD. Systematic reviews consolidating various studies confirm the link between epicardial fat thickness and the presence and severity of NAFLD [13,14], including distinctions between different stages of liver fibrosis [15-17]. Exploring this relationship further in the context of organ donation is warranted.

Regarding the utilization of steatotic grafts in deceased donor liver transplantation (DDLT), there is no standardized guideline. However, consensus suggests that mild to moderate (<30%) macro-steatotic livers typically yield favorable outcomes, with steatosis levels up to 50% deemed acceptable in DDLT [18]. Strategies to mitigate risks associated with moderate-severe hepatic steatosis in DDLT involve reducing ischemia time (e.g., <6 hours), employing de-fatting techniques (e.g., machine perfusion), refining donor-recipient matching (e.g., assigning fatty livers to low native Model for End-Stage Liver Disease (MELD) score patients), optimizing perfusion conditions such as NRP, and utilizing scoring systems.

Unfortunately, some of the newer techniques like machine perfusion or normothermic regional perfusion (NRP), combined with the gold standard diagnostics such as histopathology, are costly and may not always be practical for routine clinical implementation. Additionally, biopsies and pathological readings could lead to extended cold ischemia times, a scenario that should be avoided with fatty livers. However, in cases where the OPO and transplant centers are informed ahead of donation that the graft presents with more than 30% large droplet or macro-steatosis, the selection of the appropriate center and recipient becomes exceptionally critical.

The non-transplant hepatic radiologic literature has demonstrated that > 30% steatosis can be accurately evaluated by non-contrast CT using HU. The HU is calculated by the computer when the radiologist identifies the correct structures, without additional cost. Historical CT scans of the patient within the last three to six months before donation can be considered for assessment if current imaging is unavailable. We recommend that all potential liver donors undergo a comprehensive CT evaluation including HU measurements of the liver and spleen, liver/spleen ratio, and liver-spleen attenuation difference in the radiologist's report. This information should be clearly highlighted within the electronic donor chart for easy reference.

There were obviously limitations to the study. The study is constrained by a small sample size and a retrospective dataset, hampering the ability to draw statistically robust and definitive conclusions. However, existing evidence in radiology literature provides substantial support for this study. Another limitation is the absence of control over the pathology evaluations. Employing only pathology reports without expert evaluation by a hepatic organ donation histopathology specialist limits the strength of our recommendations.

## Conclusions

Based on our finding we would consider, first if HUL > 40, liver-to-spleen attenuation ratio (HUL/HUS) > 1, and delta HUL-HUS > +1 indicate mild to no steatosis, the OPO can proceed to donation. Second, if HUL < 40, HUL/HUS < 0.8, and delta HUL-HUS < -1 indicates at least moderate steatosis, accepting transplant centers should be alerted early in the donation offer process allowing for steatosis protocols to be activated which include considering liver biopsy and preparation for machine preservation. Third, in cases of a mixed picture, suggesting some degree of moderate steatosis, radiologic evaluations indicating steatosis should prompt immediate notification by the OPO to transplant centers, enabling a swift protocol matching steatotic livers with compatible recipients and preservation methods.

This proactive approach for all groups, diminishes organ wastage and incidence of DGF by optimizing protocols for steatotic livers, leading to cost savings within the healthcare system and ultimately enhancing patient outcomes. For instances classified as Group 3 (mixed picture), a pre- or intra-operative liver biopsy is strongly recommended. Future advancements include 1. implementation of AI prediction techniques to identify predictors of a steatotic liver suitable for transplantation, utilizing pre-donation information comprehensively and 2. utilizing AI technology to analyze CT and biopsy images to estimate the degree of steatosis accurately.
